# The Development of a Data Collection and Browser Fingerprinting System

**DOI:** 10.3390/s23063087

**Published:** 2023-03-13

**Authors:** Kiu Nai Pau, Vicki Wei Qi Lee, Shih Yin Ooi, Ying Han Pang

**Affiliations:** Faculty of Information Science and Technology, Multimedia University, Melaka 75450, Malaysia

**Keywords:** browser fingerprints, attributes, data collection, JavaScript

## Abstract

The urgent need to protect user privacy and security has emerged as the World Wide Web has become an increasingly necessary part of daily life. Browser fingerprinting is a very interesting topic in the industry of technology security. New technology will always raise new security issues and browser fingerprinting will undoubtedly follow the same process. It has become one of the most popular topics in online privacy because, to date, there is still no exact solution as to how to stop it entirely. The majority of solutions just aim to reduce the likelihood of obtaining a browser fingerprint. Research on browser fingerprinting is unquestionably required since it is essential to educate users, developers, policymakers, and law enforcement about it so that they can make strategic choices based on knowledge. Browser fingerprinting must be recognised in order to defend against privacy problems. A browser fingerprint is described as data gathered by the receiving server to identify a distant device, and it is different from cookies. Websites frequently utilize browser fingerprinting to obtain information about the type and version of the browser, as well as the operating system, and other current settings. It has been known that even when cookies are disabled, fingerprints can be used to fully or partially identify users or devices. In this communication paper, a new insight into the challenge of browser fingerprint is encouraged as a new venture. Thus, the initial way to truly understand the browser fingerprint is the need to collect browser fingerprints. In this work, the process of data collection for browser fingerprinting through scripting, to offer a complete all-in-one fingerprinting test suite, has been thoughtfully divided into appropriate sections and grouped with key information to be carried out. The objective is to gather fingerprint data with no personal identification information and make it an open source of raw datasets in the industry for any future research purposes. To our best knowledge, there are no open datasets made available for browser fingerprints in the research field. The dataset will be widely accessible by anyone interested in obtaining those data. The dataset collected will be very raw and will be in the form of a text file. Thus, the main contribution of this work is to share an open dataset of browser fingerprints along with its collection methodology.

## 1. Introduction

Browsers are the gateway to the internet and people have a tendency to spend an increasingly long time online, thus resulting in web usage growing, which will increase privacy complications. The entrance to the internet is through a browser; thus, each website needs to understand the browser and its surroundings to offer rich, fulfilling, and aesthetic services. Modern browsers freely offer websites with detailed information about the hardware and software configuration through various APIs and technologies. This allows websites to be able to fully utilize the user’s resources. Information is more prone to leaking as the size of data increases since it is harder to control; consequently, web security is becoming more challenging [1]. Most of the common websites will only request the necessary information to deliver their services, but the beast that lurks in the bushes will be requesting as much information as possible. This will cause the attackers to be able to take advantage of slight system discrepancies between users’ systems. However, through the use of web browser fingerprinting, advertisers and anti-fraud firms have discovered a new method of web tracking. A browser fingerprint is described as data gathered by the receiving server to identify a distant device [2]. When a user visits a website, browser fingerprinting involves gathering information about the settings of the user’s browser and system. Websites frequently utilize browser fingerprinting to obtain information about the type and version of the browser, as well as the operating system, IP address, and other current settings. It has been known that even when cookies are disabled, fingerprints can be used to identify particular users or devices, fully or partially. Though many mistakenly think cookies are browser fingerprints, they are different. The difference between both is that cookies are only accessible to the website from which they were obtained and cannot be shared from one website to another, whereas browser fingerprinting can track users throughout the internet. Cookies can be readily removed by adopting privacy features that affect cookie tracking. On the other hand, limiting fingerprinting is far more difficult because it is much harder to detect when it is being used, and most of the systems do not currently have a good, predictable way of preventing it.

In this work, the purpose is twofold: to present an approach to browser fingerprinting, with an in-depth understanding of attributes, which is completely transparent and with no personal identification information (PII); and that anyone new to this research area can obtain the raw datasets. This research attempts to offer a thorough, all-in-one fingerprinting test suite that is neatly divided into appropriate sections and groups’ relevant information. The data collection and attributes collected will be an open discussion and anyone can obtain it for further usage for research purposes. Most of the browser fingerprinting tools are big-scale commercialized dataset tools in a form that has never been made public, thus hindering the research conducted in this field. To fill the gap, a data collection process and a set of collected browser fingerprints will be shared in this paper. Existing browser fingerprinting tools can be seen in the studies of Eckersley [3] and Laperdix [4], which will be further discussed in Section 3. The aforementioned data collection methods are large-scale and not suitable for research purposes as there are too many fingerprints collected in a commercialized and customized way, which leads to bias in the data collected. Thus, in this paper, the data collection is collected in a non-biased way and on a small scale which is suitable for further research on browser fingerprinting. Browser fingerprinting research will be heavily adopted since any modern technology could be targeted by attackers as a weapon for destroying privacy [5]. One of the fundamental values, the right to anonymity, may be violated if users are identified without their knowledge or consent. 

Thus, research into browser fingerprinting will be encouraged; therefore, the very first step is to collect data on browser fingerprinting which is designed for open research. In this paper, the method of data collection of browser fingerprinting information is discussed, and the raw datasets are also to be shared and published for open research purposes. 

In summary, this paper provides the following contributions:

- Building a website to carry out browser fingerprint data collection which is described in Section 4;
- An in-depth understanding of the key attributes of browser fingerprinting. A total of 15 attributes have been collected and the methods to obtain those attributes are discussed in Section 5;
- The datasets of the collected browser fingerprint will be shared and downloadable via the developed website. This research has been collected for more than 500 fingerprints and the collection is still ongoing; the YTD datasets are accessible via the link presented in Section 4.

## 2. Justification of Study

Browser fingerprinting occurs when a user visits the designated website, and it is then used to gather information about how the user’s system and browser configurations. This procedure can make remarkable amounts of information about a user’s software and hardware environment available, and it can be used to create a special identity termed a browser fingerprint. Thus, the need to classify the intentions of browser fingerprinting becomes very important. Identification is necessary for the website, but the information should not be used for any possible user profiling or tracking. As such, the classification of browser fingerprints is important in the technology field and even in our everyday browsing. With that in mind, research into browser fingerprints should be increased to prevent any further privacy ramifications. To encourage the research of browser fingerprinting, this paper aims to release a public browser fingerprints dataset (with the contributors’ agreement and all profiling information anonymized), along with the mechanism on how to collect it so that the methodology can be re-adopted by researchers. The collection of browser fingerprint data is one of the crucial steps in this research field to further explore the types and associated risks of browser fingerprints. Since most of the existing datasets are not commercialized and not open, this is hindering the research opportunities in this field. Therefore, the objective of this paper is to present a data collection framework that can gather pertinent information from users’ web browsers after they request a particular internet site, which can then be merged to create a browser fingerprint. In addition, the collected datasets were also made available and open for downloading, which will be further discussed in Section 4. 

## 3. Background

In this section, the discovery of browser fingerprints will be heavily investigated as we can see that Mayer had considered whether discrepancies in origins on the internet could lead to the deanonymization of web clients [6]. Mayer was particularly interested in determining if variances in surfing environments could be used to identify users through a distant server. Mayer et al., realized that there was a browser on the testing computer which potentially displayed “quirkiness” caused by the operating system, hardware, and browser configuration. To substantiate the hypothesis, an experiment had been conducted by analysing the information collected from a browser, including the components of navigation, screen, and other HTML sources such as *navigator.plugins* and *navigator.mimeTypes* which can be extracted directly from the web page source. The result shows that 1278 (96.23%) of the 1328 clients can be individually identified [6]. However, due to the limited and restricted sources, it is hard to reach a broader conclusion based on the experiment itself. Eckersley et al. [3] proposed that the Panopticlick experiment was carried out a year later by the Electronic Frontier Foundation (EFF). In just two weeks, the experiment collected 470,161 fingerprints through communicating on social media and famous websites. Unlike Mayer et al. [6], the number of fingerprints collected provides a considerably more precise view of the situation of device variety on the web. It shows that 83.6% of fingerprints were unique, using data from HTTP headers, JavaScript, and plugins such as Flash or Java. This number increased to 94.2% if users enabled Flash or Java, as these plugins provided extra device information. The phrase “browser fingerprinting” was developed in this study, and it was the first study to demonstrate that the research is possible to be conducted on a big scale. Given that a device with a less-than-common setup can quickly be detected on the internet, privacy concerns become another rising issue. 

A browser fingerprint is a compilation of several user-device data, including hardware, operating system, browser, and configuration [7]. Browser fingerprinting is the act of gathering data from a web browser to create a device’s unique fingerprint. As a result of these attributes, an analogy with a digital fingerprint is made and it is often unique. The final fingerprint will not change even if either the VPN is activated or the user enters incognito mode. This approach can make remarkable amounts of data about a user’s software and hardware circumstances available, and it can be used to create a special ID known as a browser fingerprint in the end. Due to the potential for user tracking, privacy considerations are considerable. The threat to privacy is serious, as can be seen from the studies of Acar et al. [8] and Nikiforakis et al. [9], who adopted browser fingerprinting. Large companies such as Google indirectly advertise their adoption of a privacy policy, indicating that they use technologies to identify their users’ browsers or even devices, which leads to the conclusion that browser fingerprinting is part of their identification technologies. Browser fingerprinting has, without a doubt, become one of the privacy threats to web users because when a user visits a website, browser fingerprinting is used to gather information about how their system and browser are configured. In the end, a unique identity can be created using this procedure, which can also expose a surprising amount of information about a user’s software and hardware settings [10]. The privacy implications are significant because users can then be tracked using these fingerprints. Other than that, collecting browser fingerprint information can also be separated into negative usage and positive usage [10]. A negative usage means that unknown third parties may want to track a user without their knowledge or attack their target’s device by utilizing a known security flaw. On the other hand, the positive usage can happen if a device is out of date; users might then be alerted through recommendations for specific updates by confirming that a device is authentic and already recognized by the system, and the security of online services can also be strengthened. Browser fingerprinting can lead to a form of tracking users if used in a negative form. Thus, the concerns about privacy are crucial since browser fingerprinting can identify a device on the web in a unique way. A third party can identify a person and link his browsing behaviours within and between sessions by gathering browser fingerprints. Most significantly, because the tracking scripts are silent and run in the background, the user is oblivious to the data collection process, which is fully transparent. Browser fingerprinting can be vulnerable, which causes privacy issues; on the other hand, it can also help to improve the security of a browser. There are various ways in which browser fingerprinting can improve web security [10]. With the help of the browser fingerprint and the dataset that had been collected, patching vulnerable systems can be carried out. This means that browser vulnerabilities could be discovered in order to be fixed. Another privacy threat that can be overcome is fraud prevention. This is because verifying the actual content of a fingerprint is another way of using browser fingerprinting to increase web security. Due to the interdependencies between the attributes that have been collected, it is feasible to determine whether a fingerprint has been altered or if it fits the device that it is meant to belong to. In 2010, the use of browser fingerprinting techniques to stop online fraud was announced by ThreatMetrix, a security firm that specializes in the validation of online transactions [11]. They mentioned that scammers alter their IP addresses, remove cookies, and randomize device attributes using botnet scripts. However, depending solely on cookies to validate an online transaction is no longer sufficient since a lot of security companies such as Distil Networks, Pixel Scan, MaxMind, Sift Science, and others also implemented browser fingerprinting to detect bots and odd behaviour in their surroundings. Browser fingerprints also serve the purpose of being a website’s authentication procedure to confirm a user’s identity. Verification might take place at the time the user inputs their credentials or throughout the user session. The security advantages offered using browser fingerprinting in an authentication context were examined and evaluated in this context by numerous studies. An approach was proposed to enhance the session security with browser fingerprinting by [12]; their approach works in a way that can be described in a few steps. Firstly, the server determines whether the session cookie given by the browser is connected to an already-running session. Next, basic static fingerprinting attributes are checked by the server, including the HTTP headers, their sequence, and the IP address range. The next step will be that the client browser is prompted by the server to check for features such as CSS and WebGL. After that, the server receives from the browser a list of tested features and the system will validate the data sent by the browser. However, their method leaves users open to man-in-the-middle or cross-site request forgery (CSRF) attacks while protecting users against session hijacking [10]. Additionally, they put the suggested solution into operation without giving it a proper evaluation. All in all, the positive attributes in browser fingerprinting for enhancing web security can be seen improving gradually, and we hope that more research will carried out in the future.

### 3.1. Previous Studies

In the past few years, there have been experiments of browser fingerprint collection in large-scale studies. The three popular state-of-the-art studies include the Panopticlick [3], AmIUnique [4], and Hiding in the Crowd [7]. The experiments mentioned above have been a great influence and escalated the research of browser fingerprinting.

#### 3.1.1. Panopticlick

This experiment was conducted by Peter Eckersley [3] from the Electronic Frontier Foundation (EFF) in 2010. Over two weeks, between the 27th and the 15 February 2010, he collected approximately 470,161 fingerprints by interacting on social media and some well-known websites including Slashdot, BoingBoing, Lifehacker, Ars Technica, and io9 [3]. He noticed 83.6% of unique fingerprints used information from HTTP headers, JavaScript, and plugins such as Flash or Java. This percentage increased to 94.2% if users enabled Flash or Java because these plugins offered extra information on the device. The phrase “browser fingerprinting” was first used in this study to demonstrate that it was a massively expanded reality. The privacy issues that resulted from it are quite effective because a device with an uncommon configuration can be quickly found on the internet. The list of plugins, the list of fonts, and the user-agent were at the time the most sensitive attributes [3] The collected dataset can be found on their Github https://github.com/EFForg/cover-your-tracks (accessed on 1 October 2022) and it is open to the public. The datasets will be stored in their public database. They also mentioned that their datasets are quite biased because the data obtained are from users who care for their privacy over the internet. The population of those users performed a few simple measures, such as restricting cookies or maybe utilizing proxy servers for sensitive online browsing [3]. 

#### 3.1.2. AmIUnique

AmIUnique was conducted by Laperdrix et al. [4] in 2014. The datasets were collected between November 2014 and February 2015, and comprise approximately 118,934 fingerprints. First, they validated Eckersley’s 2010 findings [3], which indicated 89.4% unique fingerprints. However, they observed the differences in the salient attributes that make a fingerprint unique. Some of the salient attributes which can uniquely identify a fingerprint are no longer dependable. Fonts and plugins were the top reason for unique fingerprints; however, that is no longer the case as plugins have been disabled in many modern browsers as of 2018 [13]. In this study, around 17 attributes were examined. AmIUnique is considered one of the most successful research projects on fingerprints because it is the first collection that applies the technique of canvas fingerprinting. The results from this newer technique are quite powerful because they reported a significant entropy in the values gathered. Browser identification on these devices is largely dependent on HTTP headers and HTML5 canvas fingerprinting [10]. The project also analysed the variations between fingerprints from computers and mobile devices. In the rising era of smartphone proliferation, they have then proven that mobile fingerprinting also exists devices other than desktops. In their observation, only 81% of mobile fingerprints were unique, compared to 90% of computers’ fingerprints [13]. This disparity was mostly caused by the low entropy of the list of fonts and the list of plugins on mobile devices. Using their dataset, researchers simulated the effects of potential technical developments on browser fingerprinting to enhance privacy. However, they learned that uninstalling plugins and using generic HTTP headers can lower the uniqueness of desktop fingerprints by a powerful 36% [4].

#### 3.1.3. Hiding in the Crowd

Hiding in the Crowd was conducted by Gómez-Boix et al. [7] in 2018 and they successfully collected around 2,067,942 fingerprints from one of the top 15 French websites, which, according to the Alexa traffic rank, are on two specific websites. The specific websites are the weather forecast page and one of the political pages [7]. As 33.6% of the fingerprints in their dataset were found to be unique, their findings add a new level of understanding to the topic. The gap is even more pronounced when mobile devices are considered because only 18.5% of fingerprints from mobile devices were distinctive compared to 81% shown by Laperdrix et al. [4]. 

Over the years, fingerprints were collected by targeting people who are cautious of online privacy or who are concerned about privacy. With that in mind, this experiment’s data are collected based on a commercial website where anyone can be involved. The keys to recognizing the variations in fingerprint uniqueness are related to this aspect of the dataset, which has led to the enormous quantity of fingerprints collected. Additionally, canvas on mobile devices and plugins on desktops are the most recognizable features. On mobile devices, fingerprints with distinct canvas values account for 62% of all unique fingerprints; however, on personal desktops, fingerprints with distinct plugin combinations account for 30% of all unique fingerprints [7].

### 3.2. Previous Studies vs. the Proposed Research

The previous sections concerned the background of the three most widely known studies, which are Panopticlick [3], AmIUnique [4], and Hiding in the Crowd [7]. It is clear that all three studies have their uniqueness and how all of them contributed to the research of browser fingerprints. With that in mind, this section will be showing how these previous studies differ from the proposed research. The main difference is in the number of data that will be collected. All three of the studies collected fingerprints on a large scale. AmIUnique [4] collected around 118,934 in the year 2016 [4]. On the other hand, Panopticlick [3] collected around 470,161 fingerprints in 2015 [3]. Hiding in the Crowd [7] collected the greatest number of fingerprints among the three studies, which is 2,067,942, in 2018 [7], while the proposed website collected fingerprints on a smaller scale, which was carried out in the setting of the faculty of Information of Technology at the Multimedia University. A total of 600 fingerprints had been collected as of January 2023. Both the AmIUnique [4] and Panopticlick [3] datasets were biased because both websites are focused on fingerprinting, and the visitors were curious about internet monitoring, which will thus affect the precision of the results. Although Hiding in the Crowd [7] did not produce a biased dataset, which the privacy issues had raised, the project collected fingerprints through 15 French websites, which are on two distinct domains, according to the Alexa traffic rank. The clearly defined websites were a politics website and a weather forecast website [7]. Their findings were remarkable because 33.6% of the fingerprints in their sample were discovered to be unique; however, they did not have consent for collecting those fingerprints, which was considered to violate the privacy of the users. On the other hand, the proposed website will first ask for permission and inform the user that no other user profiling will be carried out (the screenshot of the page indicating the permission can be found in Section 4). The next contrast will be that the proposed website datasets will be fully transparent and anyone interested can obtain the datasets by filling out the Google form on the website. Those datasets will be in a raw text file, while the previous studies’ datasets are not easily accessible on the internet. 

## 4. Data Collection

**Figure 1:**
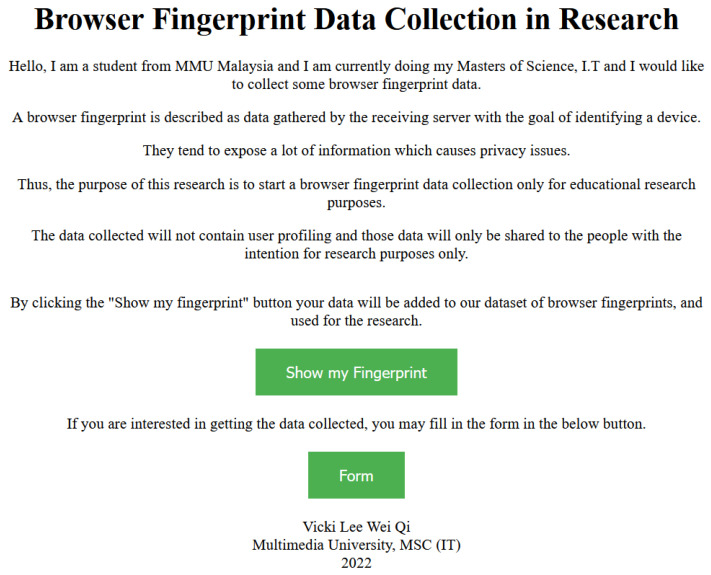
Snapshot of the first page of the designated website.

Inspired by the previous studies, it is important to know that some attributes will be very useful to profile a browser fingerprint. The attributes could be also changing along with the development of web browser settings. Thus, it is always important to re-collect the modern attributes so that their distinctive functions can be further analysed and investigated. The contributions of this data collection are two-fold. Firstly, the dominating attributes can be unearthed. Secondly, these attributes can be fine-tuned if privacy concern is in place. Thus, the purpose of this research is to develop a browser fingerprinting website, which is hosted at https://fpting.com/ (accessed on 1 December 2022) (and a snapshot of which can be viewed in Figure 1 below). The website will first require the user’s consent to collect their fingerprints and brief them about how no other user profiling will be done.

The website was built in 2022 with the purpose of collecting browser fingerprints. The collection was carried out over two months. The website is mostly built in JavaScript and the relevant component of the client-side software was inspired by the TorZillaPrint (Arkenfox). The website used different techniques to retrieve the information about the client’s web browser that will be needed for the in-depth research on browser fingerprints.

The data information from the client’s web browser will be collected once the user has visited the webpage by clicking the link. However, because of ethical reasons, consent from the user is important; thus, no personal information will be collected. Next, the server will start collecting HTTP headers as soon as the user establishes a connection to the page containing our fingerprinting script. The browser will then execute the script that gathers most of the fingerprint data if the user has not disabled JavaScript. The snapshot of the designated website will be shown in the figure below.

**Figure 2:**
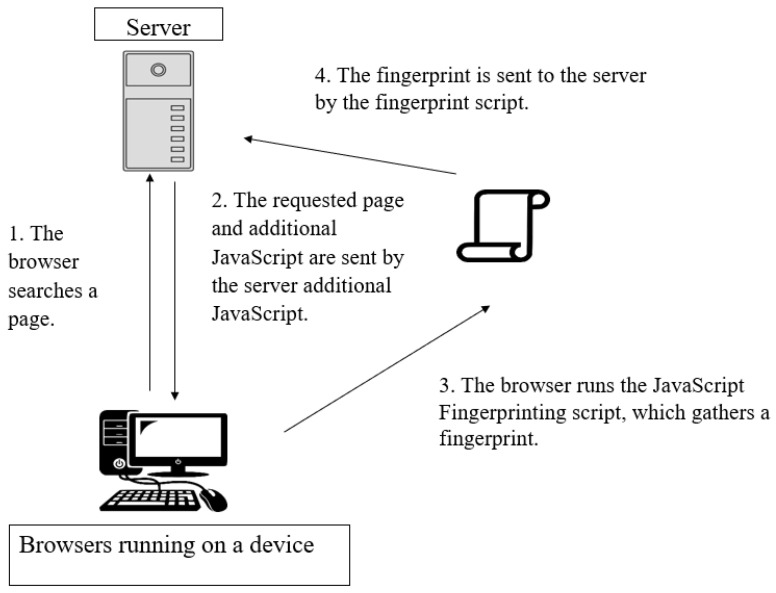
Summary of the browser fingerprinting procedure.

The script can generate a fingerprint in a few hundred milliseconds. Each fingerprint has different information depending on the browser being used, how it is configured, and the hardware and software environment it is running in. Fingerprints collected will be able to be distinguished between one browser and another. Figure 2 provides a summary of the browser fingerprinting procedure. A user’s browser sends a GET request to the server to obtain a page whenever he/she accesses a website and the server then replies with a response providing the page’s content after the request is received. The HTML returned includes fingerprinting scripts in the format of JavaScript files. These scripts may be used by the site visited as first-party scripts or through any of the third-party sites to monitor users across various websites. After the script has loaded, it will be collecting the different attributes as a whole browser fingerprint. The JavaScript script used to perform the fingerprinting must send the data it has acquired to a server after it has finished running. While some fingerprinting scripts send the entire list of attributes, others only compute a hash that is sent.

Next, the uniqueness of each browser can be distinguished by collecting the attributes of each browser. Table 1 below shows examples of collected attributes in this research. Table 1 displays the types of popular attributes to be collected, the source to retrieve the attributes from, and the example values of the attributes.

A browser fingerprint can be defined as a collection of attributes retrieved from the respective browser. Thus, the collection of attributes made up the majority of fingerprint collection. To start the data collection in this research, studying and understanding the attributes well is needed. Attribute collection in this research can be performed through the JavaScript commands with a few scripts.

## 5. Attributes

In this section, the attributes that represent the entire process of browser fingerprint collection in this research will be discussed. For this research to collect the attribute of the browser fingerprint, the methods of collecting attributes need to be seriously considered in order to create a unique browser fingerprint that distinguishes one browser from another. The details of each of the attributes will be explained in further detail as each of the attributes could be nothing on their own but will create a bigger picture when they are combined. Most of the attributes are easily able to be retrieved via scripting. All the attributes listed below never invade the privacy of the user, such as the IP address, which reveals the private information of the users. For the fingerprint attributes in this research to be more precise, two of the properties will be needed [13].

1. Uniqueness: The attributes collected in this research are individually unique but the combination of all attributes will turn them into one whole browser fingerprint. The fingerprints need to be unique to differentiate one browser from another. The fact is, if multiple browsers share the same fingerprints, they are invulnerable to browser fingerprinting tracking.
2. Stability: Because the browser fingerprint is dynamic, tracking requires a certain level of consistency even when the attributes collected are all unique [14]. Browser fingerprint evolves as a result of user setting changes, browser updates, and other occurrences. In this research, an extension randomly chooses the value of the collected canvas attributes with any of the visits; thus, the browser fingerprint will continue to be unique simply because the canvas is unique. However, it becomes difficult to monitor the fingerprint over time because the canvas is constantly shifting. This research does not collect data that do not have stability such as the viewport size, the performance, and the network speed.

In this research, the attributes collected for the data collection do not require any personal identification information of the browser user, such as the browser IP address. The privacy of the browser user will be taken seriously as the purpose is not to track the user but to understand the browser’s behaviours. In this research, the collected attributes are all non-privacy invasions as all the collected attributes do not directly invade the privacy of the user. Although each of the attributes collected may not be deemed unique, the uniqueness of browser fingerprinting will only be formed when all the attributes are collected and formed as one.

### 5.1. Triggers

Triggers act as the way each attribute is being retrieved and called in this research. There are three types of triggers: automatic, context-dependent, and user-triggered. This section will present the ways in which attributes in this research can be retrieved. Table 2 shows how the values of the attributes in this research are collected based on triggers. Specific values such as browsers, OS, and many more are changed automatically, with no involvement from the user. The first column shows the attributes collected, the second column the type of triggers and the last column the values of the attributes.

How to obtain the attributes in this research will be explained below. The types of triggers are the following [15]:

1. Automatic: This event occurs automatically without the user’s permission. For example, when the user-agent, browser, or OS attribute is collected, there is no need for the user’s setting changes because most of the values are fixed [13].
2. Context-dependent: This is happening because of the user’s settings/concept and due to how the user configures their personal devices settings. For example, attributes collected such as the monitor resolution, location, or time zone can easily be modified by the user as the values are not fixed.
3. User-triggered: It requires user involvement input on some attributes. For example, the scenario of how the user enables cookies on their browser.

Furthermore, the attributes collected in this research can be classified into a few main groups. They consist of the HTTP headers field, and attributes collected using JavaScript and Flash. All these attributes can be collected in specific ways and each of them is unique.

### 5.2. HTTP Headers Fields

A portion of the information sent with an HTTP request is useful for the server as regards understanding the type of device making the request, how the response should be sent, or the user’s preferred languages [14]. All browsers send HTTP headers, which servers can easily gather and store, making them the perfect attributes to utilize in a browser fingerprint. To illustrate the above statement, the user-agent attribute collected in this research will be retrieved via the HTTP header. The user-agent header contains several useful fingerprinting details, the information about the hardware or the engine used to include the operating system, the browser, and its version [13]. Table 3 below shows the list of the user agents that can be found in the datasets that had been collected. The first column the value of the user-agent header and the other column shows the source of the browser.

### 5.3. Java Script Attributes

This section will be discussing how and which attributes will be collected using JavaScript. Most of the attributes will be collected via JavaScript [14]. This is because browsers offer a wide range of APIs that reveal information about the user’s device to help developers adapt websites to the user’s device. For instance, altering the browser’s screen size will result in the browser leaking several APIs that will divulge user information. Canvas and the audio API, two resources that are readily available without any type of authorization, entail the collection of extremely distinctive fingerprinting characteristics via fingerprinting.

#### 5.3.1. Navigator Object

The navigator object is the unique object exposed by default in all major browsers; it can be used to retrieve several properties that offer details about the OS and the browser [12]. There are various types of navigator objects depending on their purpose. The examples consist of the *navigator.plugins*, *navigator.languages*, *navigator.doNotTrack*, *navigator.oscpu*, etc. For example, in this research, the *navigator.platform* will be returning to the platform the browser is running on. Even though the OS listed in the user-agent header is duplicated in this information, it can be used to determine whether the OS was indeed altered. Table 4 below shows the list of platforms which is retrieved from the datasets collected in this research.

On the other hand, *navigator.cookieEnabled* used in this research will be indicating the value that indicates whether the browser has enabled cookies or not. If the user has enabled them, it will show as true, while if the user disabled them, the output will be false.

#### 5.3.2. Window and Screen

The attribute of the window and screen properties in this research is collected via JavaScript. Through the screen and window objects, the browser exposes several characteristics that correspond to the size of the screen and consider the window. All the definitions of the attribute that provide the information of the screen and window will be discussed in Table 5 below. The table shows the collected window and screen attribute which is further grouped into more detail. Table 5 has three columns: the first column shows the example of the collected window and screen attribute, the second column shows the possible values for the attribute, and the last column shows the description of each attribute that had been collected.

#### 5.3.3. Canvas Fingerprinting

**Figure 3:**
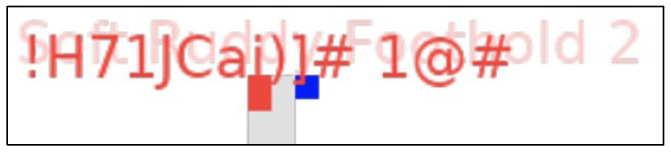
Canvas fingerprint generated with Akamai Bot Manager fingerprinting script.

**Figure 4:**
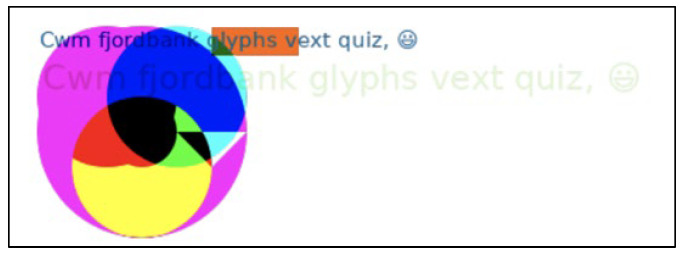
The rendered image in Fingeprintjs2′s canvas fingerprinting.

Canvas is an HTML5 API employed to generate visuals and animations on a web page using JavaScript scripting. Canvas fingerprinting [16] is known to provide a highly unique value to fingerprinting and can be used for online tracking purposes. The method is based on the idea that different computers may render the same canvas image differently. There are different causes for this. The final images may have a different checksum even though they are pixel-identical because web browsers implement various image-processing engines, export settings, and compression levels [16]. Operating systems use a variety of typefaces, anti-aliasing algorithms, and sub-pixel rendering settings at the system level [16]. Thus, in this research, it is important to collect the attribute of the canvas fingerprint. The canvas fingerprinting approach works based on the same concept as the preceding example, which is that the different systems (browsers) draw a graphical item differently, resulting in different fingerprints. Mowery and Shacam [17] show that the display of fonts and graphical elements differs somewhat between browsers, which allows a distinct signature to be extracted. Figure 3 and Figure 4 below show the canvas generated with Akamai and PerimeterX fingerprinting scripts.

In this research, to create a signature from the canvas, the pixels were required to be exported from the application’s memory using the *toDataURL()* function. The binary image file’s base64-encoded string will then be returned. The output of the canvas fingerprint property is then gathered after creating an MD5 hash of this string or even extracting the CRC checksum from the IDAT chunk that is located between bytes 16 and 12 at the end of every PNG file. A canvas fingerprint can be created using a variety of techniques. Drawing a blank rectangle and adding different functions such as coloured lines, overlays, and anti-aliasing filters will still be the fundamental idea. Algorithm 1 demonstrates the script on how a canvas fingerprint can be generated.
**Algorithm 1** How the canvas fingerprint is being generated. <b>Hash:</b> <span id=‘hash-code’></span><br> <canvas id=‘myCanvas’ width=‘200’ height=‘40’ style=‘border:1px solid #000000;’></canvas> <script> var canvas = document.getElementById(“myCanvas”); var ctx = canvas.getContext(“2d”); ctx.fillStyle = “rgb(255,0,255)”; ctx.beginPath(); ctx.rect(20, 20, 150, 100); ctx.fill(); ctx.stroke(); ctx.closePath(); ctx.beginPath(); ctx.fillStyle = “rgb(0,255,255)”; ctx.arc(50, 50, 50, 0, Math.PI * 2, true); ctx.fill(); ctx.stroke(); ctx.closePath(); txt = ‘abz190#$%^@£éú’; ctx.textBaseline = “top”; ctx.font = ‘17px “Arial 17”‘; ctx.textBaseline = “alphabetic”; ctx.fillStyle = “rgb(255,5,5)”; ctx.rotate(.03); ctx.fillText(txt, 4, 17); //hashing function src = canvas.toDataURL(); hash = 0; for (i = 0; i < src.length; i++) {         char = src.charCodeAt(i);         hash = ((hash<<5)-hash)+char;         hash = hash & hash; } //output this however you want $(‘#hash-code’).html(hash); </script>

#### 5.3.4. CSS Fingerprinting

The CSS fingerprint of the browser is also collected in this research. In particular, CSS offers query strings, which are activated each time the corresponding CSS attribute is used. To determine the attributes that a web browser has applied to the web server, a query string is added to the request for content. By looking at the query string of the web request when a web browser sends a request to a web server, the latter decides how the browser will interpret the CSS description [18]. A few sub-properties of this attribute were collected, such as the system fonts, colour system, and media queries.

### 5.4. List of Fonts

Fonts are frequently gathered using the Flash plugin. One can obtain the complete list of fonts installed on the browser with just a few lines of programming. However, plugins are being phased out in favour of a feature-rich HTML5 environment in contemporary browsers due to security and stability concerns [7]. Flash was discontinued by Adobe in 2020 [19] because it primarily poses a security concern, and it is being replaced by browser extensions which offer fewer rights than plugins. This means that to access the list of fonts, a different method was required to be used in this research. A new solution was suggested by Nikiforakis et al. [9], in which he revealed that it could be possible to obtain the fonts through JavaScript. In this research, the attribute of fonts was retrieved via font enumeration instead, as most users, to date, had disabled Flash in their browsers. The purpose is to contrast the sizes of two HTML elements, one of which makes use of the system’s default font, while the other of the font that will be tested. One can determine whether the requested font was utilized or the fallback font was used by examining the *<div>* element’s dimensions. The main distinction between these two collection techniques is that although Flash provides instructions for all installed fonts at once, JavaScript requires that each font be checked separately. The downside of using font enumeration is that it is a time-consuming process and it will slow down the loading of the web page. 

## 6. Results and Discussion

**Figure 5:**
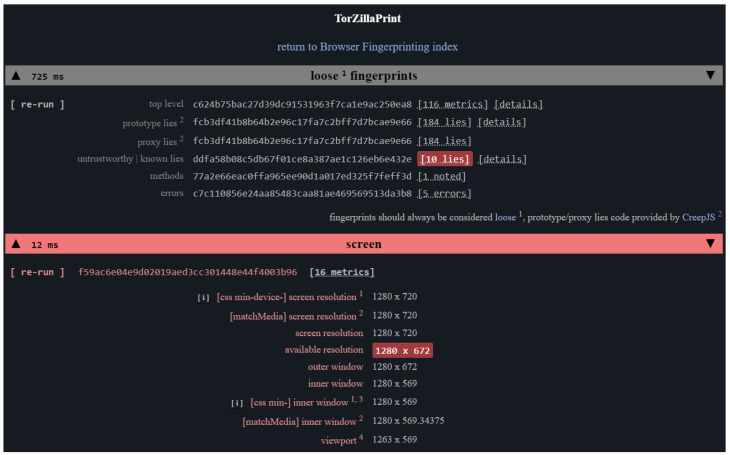
Snapshot of the website built.

In this research, the collected raw datasets will be made available to the public upon request. We postulate that this will escalate browser fingerprinting research. The data collected are in the raw state and intentionally left unprocessed so that all the fingerprints collected are original. Upon the publication of this paper, one may request a free copy of the collected datasets with proper citation of this paper. The request can be performed by sending details through Google forms on the designated website. Figure 5 presents the overview of the website built.

**Figure 6:**
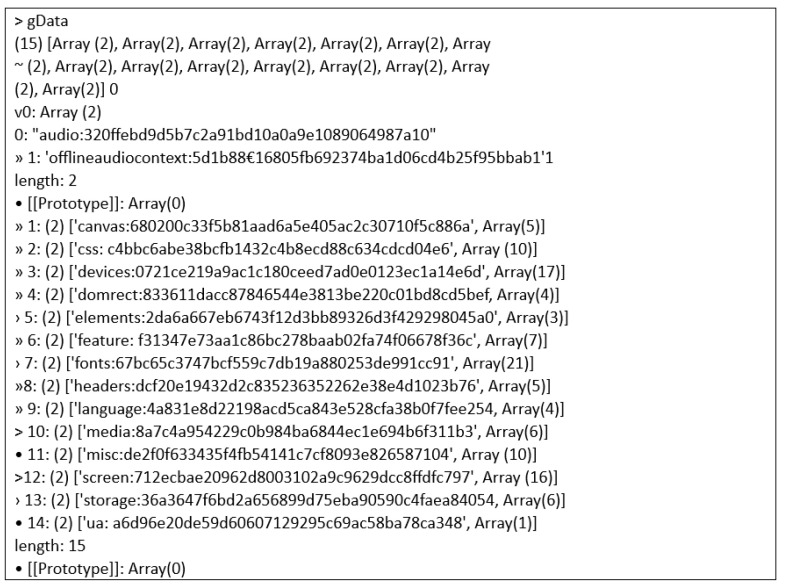
Example of attribute array values in the console log of the website.

The website consisted of two compartments which are a set of scripts that gather data from devices visiting the developed page and a web server that provides resources and keeps client information in a script. This research involved executing the JavaScript script generic.js, whose operation includes the sequential calling of functions that establish the fundamental attributes of the user’s device and the browser. Each value collected is written to an array before being processed through a hash function. Figure 6 below shows the attribute array values in the console log of the website that was called back.

**Figure 7:**
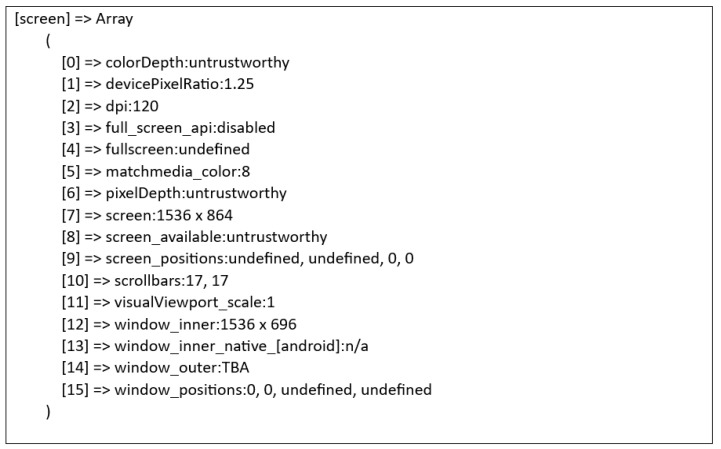
Example of screen attributes data collected in a text file.

On the other hand, Figure 7 below shows an example of the text file of the data that had been collected in this research. The figure below presents an example of data collected for the screen attribute.

**Figure 8:**
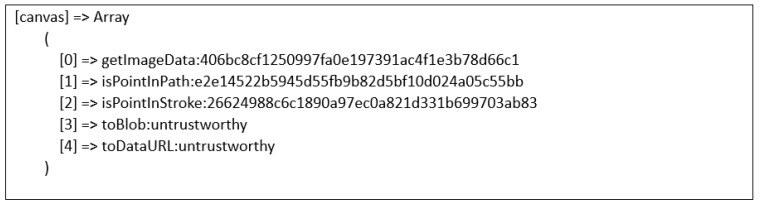
Example of canvas fingerprint attribute data collected in a text file.

Next, Figure 8 shows an example of the attribute of the canvas fingerprint that had been collected.

### Discussion over the Dataset

**Figure 9:**
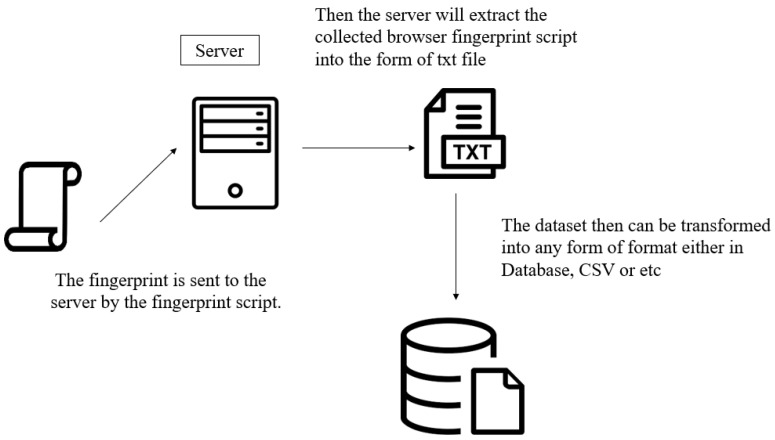
The process of the browser fingerprint data being extracted into a text file.

In this proposed website, the browser fingerprint data will be extracted into the form of a text file. In this section, the process of how the data is extracted and turned into the raw text file is discussed. Firstly, the collected fingerprint will be sent to the server via the fingerprinting script and the server will then extract the collected browser fingerprint script into the form of a text file. Most of the existing studies or research placed their data under the database; in this research, however, the reason the data will be in this form is that a text file is considered the most basic and raw form of any file. For instance, interested researchers can download those datasets in the form of a text file and convert them into a comma-separated values (CSV) file. CSV files are plain text files, which makes them easier to import into a spreadsheet or any other storage database and are able to be used on any software. A dataset in the form of CSV can be easily used by machine learning, which will be able to enhance the research into browser fingerprint in the future. This is one of the reasons this proposed research is different from the previous studies. Figure 9 shows the process of the browser fingerprint data being extracted into a text file.

## 7. Conclusions

In this study, a browser fingerprinting website has been developed and hosted at https://fpting.com/ (accessed on 1 December 2022). To date, a total of 600 fingerprints have been collected. The purpose of this research is to present a method of browser fingerprinting collection with no personal identification information. The other objective would be to collect the browser fingerprint datasets and making them available to the public. This is because there are not many existing datasets of browser fingerprints in the research field; thus, with the help of the dataset, more research on browser fingerprints will be encouraged. The dataset in this proposed website is much easier to access by everyone in the industry via the website. The researcher will just need to fill out the Google form and the set of datasets will be provided. All the data will be in a very raw form, which is in a text file format. Most of the existing studies do not provide those datasets openly on the internet. 

Browser fingerprinting may be new in the research industry, but it does raise problems about privacy and has the potential of becoming a deadly tool for attackers, just as any other modern technology [20]. It is hard to prevent or restrict the gathering of browser parameters. In the hands of an attacker, the consistency with which a browser fingerprint identifies a user can turn into a dangerous weapon [5]. Although modern technology provides a much easier and more comfortable user experience, it is unavoidable that privacy concerns will be an issue; therefore, understanding browser fingerprints is crucial. Moreover, how the attributes were collected was also discussed in depth in this paper as attributes are the main key to browser fingerprint. This is because attributes are key in browser fingerprinting, which leads to how unique one whole browser fingerprint will be. After all, it is hoped that this research will allow researchers to take a first step in analysing browser fingerprint because it has the potential to increase online security for millions of people in the real world.

## Figures and Tables

**Table 1:** Example of browser fingerprint.

Attribute	Source	Value Examples
Accept	HTTP header	Text/html, application/xhtml + xml, application/xml; q = 0.9, image/webp, */*; q = 0.8
Connection	HTTP header	Close
User-agent	HTTP header	Mozilla/5.0 (Windows NT 10.0; WOW64) AppleWebKit/537.36 (KHTML, such as Gecko) Chrome/102.0.5005.63 Safari/537.36
Canvas	JavaScript	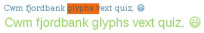
Cookies	JavaScript	Yes
Local storage	JavaScript	Yes
Time zone	JavaScript	−180
Lists of fonts	Flash or JavaScript	Abyssinica SIL, Aharoni CLM, AR PL UMing CN, AR PL UMing HK, AR PL UMing TW…

**Table 2:** Browser fingerprint examples showing attribute triggers with example values.

Attribute	Triggers	Value
Browser	Automatic	Chrome 74
OS	Automatic	Windows NT 4.0|32 bits
User-agent	Automatic	Mozilla/5.0 (Windows NT 10.0; WOW64) AppleWebKit/537.36 (KHTML, such as Gecko) Chrome/102.0.5005.63 Safari/537.36
Display font	Context	24|1536|864|1536|824
Time zone	Context	−7
Plugins	Automatic/user	N/A
Cookie	User	true
Browser	Automatic	Chrome 74

**Table 3:** Example of user-agent header value and their sources.

User-Agent Header Value	Source
Mozilla/5.0 (iPhone; CPU iPhone OS 16_0 such as Mac OS X) AppleWebKit/605.1.15 (KHTML, such as Gecko) Version/16.0 Mobile/15E148 Safari/604.1	Safari on the iPhone
Mozilla/5.0 (Windows NT 10.0; Win64; x64; rv:91.0) Gecko/20100101 Firefox/91.0	Firefox on Windows 10
Mozilla/5.0 (Windows NT 10.0; WOW64) AppleWebKit/537.36 (KHTML, such as Gecko) Chrome/102.0.5005.63 Safari/537.36	Chrome 73 on Windows 10

**Table 4:** Examples of platforms and their sources retrieved from the designated website.

Example of Platforms	Source
Linux x86_64, Linux armv7l, Linux armv8l, Linux i686, Linux aarch64	Browsers running on the Linux
iPad, iPhone	Browsers running on iOS.
Win64, Win32	Browsers running on the windows

**Table 5:** Example of screen and window attributes information.

Attribute	Possible Values	Description
screen.width	1280	Pixel width of the screen area that is exposed to the web. If there are several screens, it ought to return the value of the screen on which the browser window is now visible. The browser window’s size has no bearing on the value.
screen.avail	1024	Amount of horizontal and vertical space in pixels available to the browser window.
screen.colorDepth	24	Colour depth of the screen.
screen.dpi	120	Dots per inch is to describe the resolution of the image on the screen.
window.inner	1536	The browser’s width and height in pixels including the scrollbar’s size.

## Data Availability

The data presented in this study are available on request from the URL disclosed in this manuscript.
